# Sustained Neural Stem Cell-Based Intraocular Delivery of CNTF Attenuates Photoreceptor Loss in the *nclf* Mouse Model of Neuronal Ceroid Lipofuscinosis

**DOI:** 10.1371/journal.pone.0127204

**Published:** 2015-05-20

**Authors:** Wanda Jankowiak, Katharina Kruszewski, Kai Flachsbarth, Christos Skevas, Gisbert Richard, Klaus Rüther, Thomas Braulke, Udo Bartsch

**Affiliations:** 1 Department of Ophthalmology, University Medical Center Hamburg-Eppendorf, Hamburg, Germany; 2 Department of Ophthalmology, Sankt Gertrauden-Krankenhaus, Berlin, Germany; 3 Department of Biochemistry, Children’s Hospital, University Medical Center Hamburg-Eppendorf, Hamburg, Germany; University of Cologne, GERMANY

## Abstract

A sustained intraocular administration of neurotrophic factors is among the strategies aimed at establishing treatments for currently untreatable degenerative retinal disorders. In the present study we have analyzed the neuroprotective effects of a continuous neural stem (NS) cell-based intraocular delivery of ciliary neurotrophic factor (CNTF) on photoreceptor cells in the *nclf* mouse, an animal model of the neurodegenerative lysosomal storage disorder variant late infantile neuronal ceroid lipofuscinosis (vLINCL). To this aim, we genetically modified adherently cultivated NS cells with a polycistronic lentiviral vector encoding a secretable variant of CNTF together with a Venus reporter gene (CNTF-NS cells). NS cells for control experiments (control-NS cells) were modified with a vector encoding the reporter gene tdTomato. Clonal CNTF-NS and control-NS cell lines were established using fluorescent activated cell sorting and intravitreally grafted into 14 days old *nclf* mice at the onset of retinal degeneration. The grafted cells preferentially differentiated into astrocytes that were attached to the posterior side of the lenses and the vitreal side of the retinas and stably expressed the transgenes for at least six weeks, the latest post-transplantation time point analyzed. Integration of donor cells into host retinas, ongoing proliferation of grafted cells or adverse effects of the donor cells on the morphology of the host eyes were not observed. Quantitative analyses of host retinas two, four and six weeks after cell transplantation revealed the presence of significantly more photoreceptor cells in eyes with grafted CNTF-NS cells than in eyes with grafted control-NS cells. This is the first demonstration that a continuous intraocular administration of a neurotrophic factor attenuates retinal degeneration in an animal model of neuronal ceroid lipofuscinosis.

## Introduction

Neuronal ceroid lipofuscinosis (NCL) comprises a heterogeneous group of neurodegenerative lysosomal storage diseases of mainly childhood and youth. At present, mutations in more than a dozen different genes have been identified that cause NCL. Most of these genes encode soluble lysosomal enzymes or transmembrane proteins localized in lysosomes or the endoplasmic reticulum (ER). Other locations described for some NCL proteins include the ER-Golgi intermediate complex, the cytosol, synaptic vesicles or the plasma membrane (http://www.ucl.ac.uk/ncl/mutation.shtml) [1–5]. Despite the heterogeneity of the disease-associated genes, several symptoms are common to most of these fatal storage disorders, including progressive mental deterioration, motor malfunctions, seizures, and premature death. Loss of vision due to retinal degeneration is another characteristic symptom of several NCL forms, and has been described in CLN1, CLN2, CLN3, CLN5, CLN6, CLN7 and CLN8 patients [4, 6, 7].

Mutations in the *CLN6* gene cause variant late infantile NCL (vLINCL), or in rare cases adult onset Kufs type A disease [8]. The function of CLN6, a polytopic membrane protein of the endoplasmic reticulum (ER) with 311 amino acids and 7 predicted transmembrane domains is unknown [9–12]. Until now, 71 pathogenic mutations have been identified in the *CLN6* gene (http://www.ucl.ac.uk/ncl/CLN6mutationtable.htm), which may differ significantly in their impact on the severity, time course and the age of onset of the disease [13]. While about 50% of the affected children present an early retinal phenotype [4], the retina has been reported to be unaffected in patients with an CLN6-linked adult onset of the disease [8].

The *nclf* mouse, a naturally occurring mouse model of CLN6 disease [14], carries a c.307insC mutation in the *CLN6* gene that is also present in CLN6 patients of Pakistani origin [9, 10]. The single base insertion leads to a frameshift, resulting in a truncated CLN6 protein with a reduced half-life [15, 16]. Similar to human patients carrying mutations in the *CLN6* gene, the *nclf* mouse is characterized by an early-onset retinal degeneration. Reactive gliosis and apoptotic degeneration of photoreceptor cells becomes detectable in the mutant as early as one month after birth. Other characteristic features of the retinal phenotype of *nclf* mice include accumulation of storage material in various retinal cell types, dysregulation of several lysosomal proteins, and activation of microglial cells. Progressive apoptotic degeneration of photoreceptors in *nclf* mice is nearly complete at the end of the first postnatal year, and paralleled by progressive visual deterioration as measured in electroretinogram (ERG) recordings, optokinetic tracking experiments, and visual cliff tests [17–19].

Approaches to develop treatments for the neurological symptoms associated with NCLs include enzyme replacement therapy, gene therapy, stem cell therapy, and immune therapy [20–23]. In the retina, a delay in photoreceptor degeneration and/or deterioration of visual function has been reported after intravitreal transplantations of neural progenitor cells in a mouse model of CLN8 disease [24], adeno-associated virus- (AAV) mediated ocular gene transfer of palmitoyl protein thioesterase-1 in a mouse model of CLN1 disease [25] and attenuation of reactive microgliosis in a mouse model of CLN6 disease [18]. Given that a number of growth factors and cytokines have been demonstrated to delay photoreceptor degeneration and visual impairment in various animal models of induced or inherited retinal degeneration [26–29], neuroprotective approaches may represent another strategy to ameliorate retinal degeneration in NCL. However, neurotrophic factors do not ordinarily cross the blood-retina barrier and have short half-life times. Robust neuroprotective effects therefore depend on a sustained intraocular administration of these factors which may be achieved by viral or non-viral gene transfer to endogenous retinal cell types, or by intraocular transplantations of cells that have been genetically modified to secrete these factors [26–29].

In the present study, we have evaluated the neuroprotective effects of a sustained neural stem cell-based intraocular administration of ciliary neurotrophic factor (CNTF), a member of the interleukin-6 family of cytokines [30] that has been shown to potently rescue retinal structure in various animal models of retinal degeneration [26, 28], on photoreceptor cells in the *nclf* mouse. To this aim, we used a polycistronic lentiviral vector to generate clonally derived neural stem (NS) cell lines with an ectopic expression of a secretable variant of the cytokine. The CNTF-secreting NS cells were grafted into the vitreous cavity of *nclf* mice at the onset of retinal degeneration, and the numbers of surviving photoreceptor cells were determined at different post-transplantation time points. This is the first report demonstrating that a sustained intraocular administration of a neurotrophic factor attenuates retinal degeneration in a mouse model of NCL.

## Materials and Methods

### Animals

Mutant mice harboring the c.307insC frameshift mutation in the *CLN6* gene (B6.Cg-Cln6^*nclf*^/J; in the following termed *nclf* mice) were purchased from The Jackson Laboratory (Bar Harbor, ME). Mice were maintained on a C57BL/6J genetic background and genotyped by PCR analysis of DNA from tail biopsies and subsequent sequencing of the PCR product [9]. C57BL/6J wild-type mice were used for control experiments. All animal experiments were carried out in accordance with the German Animal Welfare Act on protection of animals and were approved by the local ethics committee (Freie und Hansestadt Hamburg—Amt für Gesundheit und Verbraucherschutz; permit number 59/12). All cell transplantations were performed under ketamine and xylazine anesthesia, and all efforts were made to minimize suffering of the animals.

### Lentiviral vectors, NS cell transduction and derivation of a clonal NS cell line with elevated CNTF expression

To generate CNTF-secreting neural stem cells (CNTF-NS cells), the mouse CNTF cDNA was ligated in frame with the Ig к-chain leader sequence of pSecTag2 B (Life Technologies, Darmstadt, Germany) and cloned into the polycistronic lentiviral vector pCAG-IRES-VENUS-2A-ZEO, encoding the internal ribosome entry site (IRES) of the encephalomyocarditis virus, a Venus reporter gene, the P2A sequence of porcine teschovirus-1 and a zeocin resistance gene under regulatory control of the cytomegalovirus enhancer/chicken ß-actin (CAG) promoter, giving rise to pCAG-CNTF-IRES-Venus-2A-ZEO. A vector containing the CAG promoter, the IRES sequence, and a tdTomato reporter gene fused to a blasticidin resistance gene (pCAG-IRES-tdTomato/BSD) was generated to establish NS cell lines for control experiments (control-NS cells). Lentiviral particles were produced by transient transfection of HEK 293T cells as described (http://www.LentiGo-Vectors.de).

To generate neural stem (NS) cell lines with high expression levels of CNTF, a previously established clonal CNTF-NS cell line [31] was again transduced with pCAG-CNTF-IRES-Venus-2A-ZEO. Positive cells were selected by cultivation in DMEM/F12 (Life Technologies) supplemented with 0.3% glucose, 2 mM glutamine, 3 mM sodium bicarbonate, 5 mM HEPES (all from Sigma-Aldrich, St. Louis, MO; in the following termed NS cell medium), 10 ng/ml epidermal growth factor (EGF) and 10 ng/ml fibroblast growth factor-2 (FGF-2; both from TEBU, Offenbach, Germany), 1% N2 and 1% B27 (both from Life Technologies) and 200 μg/ml zeocin (InvivoGen, San Diego, CA). Single cells with the highest expression level of the reporter gene in these cultures were selected using fluorescence activated cell sorting (FACS; FACSArialllu, BD Bioscience, San Diego, CA), plated into 96 well plates and again clonally expanded. For control experiments, wild-type NS cells were transduced with pCAG-IRES-tdTomato/BSD (control-NS cells). Single cells with the highest expression levels of tdTomato were selected using FACS, and clonally expanded in the presence of 4 μg/ml blasticidin (Life Technologies) to establish clonal control-NS cell lines with high expression levels of the reporter gene.

To semi-quantitatively compare secretion levels of CNTF between different NS cell clones, 0.5 x 10^6^ cells of each clonal cell line were cultivated for 24 hours in 0.5 ml medium, and equal volumes of culture supernatants were analyzed in Western blots using polyclonal rabbit anti-CNTF antibodies (1:500; Santa Cruz Biotechnology Inc., Santa Cruz, CA) and horseradish peroxidase-conjugated anti-rabbit secondary antibodies (1:15.000; Jackson Immunoresearch Laboratories, West Grove, PA). The clonal cell line with the highest expression level of CNTF was selected for further experiments, and the amount of CNTF secreted from this cell line at passage 11 and 19 was estimated in three independent Western blot analyses of culture supernatants using serial dilutions of recombinant mouse CNTF (Biomol, Hamburg, Germany) as a reference. Densitometric analysis of immunoreactive bands was performed using ImageJ software (NIH, Bethesda, MD).

### *In vitro* differentiation of NS cells and immunocytochemistry

To analyze expression of CNTF in differentiated neural cell types *in vitro*, CNTF-NS and control-NS cells were differentiated into astrocytes by maintaining the cells for 7 days in NS cell medium supplemented with 1% fetal calf serum (Life Technologies) and 2% B27. To differentiate NS cells into neurons, cells were cultivated for three days in NS cell medium containing 5 ng/ml FGF-2, 1% N2 and 2% B27, followed by cultivation for additional four days in a 1:1 mixture of NS cell medium and Neurobasal medium (Life Technologies) supplemented with 0.25% N2 and 2% B27. Cells were fixed in 4% paraformaldehyde (PA) in phosphate buffered saline (PBS; pH 7.4), blocked in PBS containing 0.1% bovine serum albumin (BSA) and 0.3% Triton X-100 (both from Sigma-Aldrich), and simultaneously incubated with polyclonal rabbit anti-CNTF antibodies (1:100) and monoclonal mouse anti-glial fibrillary acidic protein (GFAP, 1:500; Sigma-Aldrich) or monoclonal mouse anti-microtubule associated protein 2 (MAP2, 1:200) antibodies (Sigma-Aldrich) overnight at room temperature. Cy2-, Cy3- or Cy5-conjugated secondary antibodies (all diluted 1:200; Jackson Immunoresearch Laboratories) were applied for 3 hours to detect primary antibodies, and cell nuclei were stained with 4’,6-diamidino-2-phenylindole (DAPI; Sigma-Aldrich).

### Intravitreal cell transplantations and immunohistochemistry

*Nclf* mice received intravitreal transplantations of NS cells at postnatal day 14. Animals were deeply anesthetized by an intraperitoneal injection of ketamine and xylazine, and 2 μl of vitreous fluid were slowly removed from the eyes using a glass micropipette that was inserted into the vitreous cavity at the junction between sclera and cornea. Subsequently, 7.6x10^5^ CNTF-NS cells in 2 μl PBS were injected into one eye, and the same number of control-NS cells in the same volume of PBS into the contralateral eye. Care was taken to not damage the lens or the retina during the removal of the vitreous fluid or the injection of the cells. Eyes were analyzed two, four and six weeks after transplantation.

Eyes from untreated wild-type mice and *nclf* mice with grafted CNTF-NS or control-NS cells were immersion-fixed in 4% PA, cryoprotected in an ascending series of sucrose, frozen and serially sectioned with a cryostat at a thickness of 25 μm. For determination of photoreceptor numbers, central (i.e. in the plane of the optic disc) retina sections were incubated with polyclonal rabbit anti-recoverin antibodies (Millipore, Bedford, MA). To study expression of CNTF and differentiation of grafted cells, lenses with attached donor cells were simultaneously incubated with polyclonal rabbit anti-CNTF (1:100) and either monoclonal mouse anti-GFAP (1:500), monoclonal mouse anti-*ß*-tubulin III (1:1000; Sigma-Aldrich), or monoclonal rat anti-myelin basic protein (MBP; 1:200; Millipore, Bedford, MA) antibodies. Proliferation of grafted NS cells was evaluated by incubating lenses with attached CNTF-NS or control-NS cells with polyclonal rabbit anti-Ki-67 antibodies (1:200; Abcam, Cambridge, MA) one week and six weeks after transplantation (n = 6 for each cell population and post-transplantation time point). At least 1,000 CNTF-NS or control-NS cells were analyzed for expression of Ki-67 at each post-transplantation interval, and the percentage of positive cells was calculated. Primary antibodies were detected with Cy2-, Cy3- or Cy5-conjugated secondary antibodies (all diluted 1:200; Jackson Immunoresearch Laboratories), and retinal sections and lenses with attached NS cells were stained with DAPI and analyzed with an Olympus FV 1000 confocal microscope (Olympus, Hamburg, Germany).

### Photoreceptor counts and outer nuclear layer thickness

Entire central retinal sections stained with anti-recoverin antibodies and DAPI were photographed and the individual images were merged using Photoshop CS3 software (Adobe Systems Inc., San Jose, CA). Photoreceptors were counted in six defined areas (each covering the outer nuclear layer over a length of 220 μm) at positions corresponding to 25, 50 and 75% of the distance between the optic disc and the periphery of the temporal or nasal retina. Photoreceptor numbers were determined in *nclf* mice two, four and six weeks after transplantation (6 mice for each post-transplantation interval) and in one month old untreated wild-type mice (n = 6). Statistical analysis of data was performed with a mixed two-way ANOVA (having Time as between groups factor and Treatment as within groups factor) followed by Newman-Keuls post-hoc analyses.

The thickness of the outer nuclear layer (defined as DAPI-positive photoreceptor nuclei) was measured in central retinal sections at nine equidistant positions between the optic nerve head and the peripheral margin of the nasal and temporal retina, respectively. Statistical analysis of data was performed with the Student’s t-test for paired samples.

## Results

### Generation of clonal CNTF-NS and control-NS cell lines

**Fig 1:**
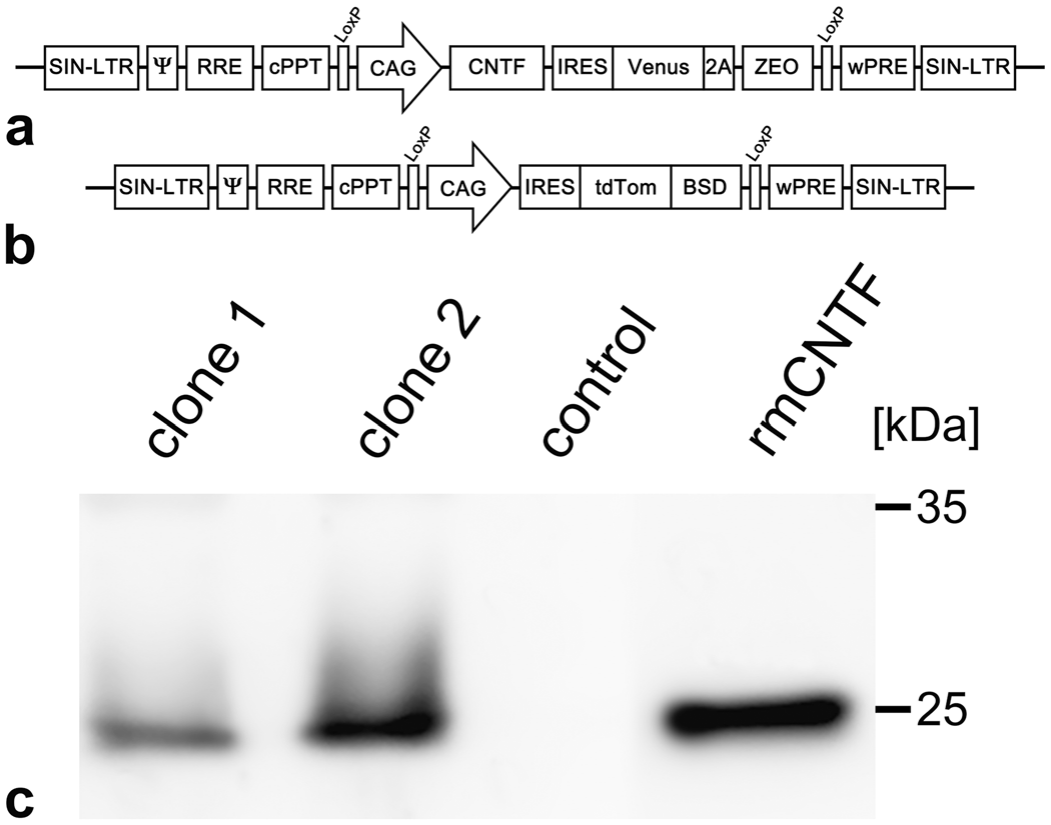
Lentiviral vectors and immunoblot analyses of culture supernatants from clonal CNTF-NS and control-NS cell lines. A lentiviral vector encoding a secretable variant of mouse ciliary neurotrophic factor (CNTF), an internal ribosome entry site (IRES) sequence of the encephalomyocarditis virus and a Venus reporter and a zeocin (ZEO) resistance gene separated by a P2A sequence of porcine teschovirus-1 (2A) under regulatory control of the cytomegalovirus enhancer/chicken ß-actin (CAG) promoter (a) was used to generate CNTF-secreting NS cells. NS cells for control experiments were transduced with a vector containing the CAG promoter, an IRES sequence and a tdTomato (tdTom) reporter gene fused to a blasticidin (BSD) resistance gene (b). Immunoblot analysis (c) of culture supernatants from the newly established CNTF-NS cell clone (clone 2) revealed elevated secretion levels of CNTF when compared to the original clonal CNTF-NS cell line (clone 1). Supernatants from control-NS cell clones (control) lacked detectable levels of the cytokine (c). Recombinant mouse CNTF (rmCNTF) was loaded as a reference. Ψ, packaging signal; cPPT, central polypurine tract; LoxP, recognition site of Cre recombinase; RRE, rev-responsive element; SIN-LTR, self-inactivating long-terminal repeat; wPRE, woodchuck hepatitis virus posttranscriptional regulatory element.

A previously established clonal NS cell line expressing CNTF together with a Venus reporter and a zeocin resistance gene (designated clone 1) [31] was again transduced with the polycistronic lentiviral vector pCAG-CNTF-IRES-Venus-2A-ZEO (Fig 1a) to generate a NS cell line with elevated expression levels of the cytokine. Cells with the highest expression levels of the Venus reporter gene were selected by FACS and clonally expanded. After five rounds of transductions and clonal expansions, semi-quantitative Western blot analyses of culture supernatants from the clonal cell lines revealed significantly increased levels of CNTF when compared to supernatants of the original CNTF-NS cell line, and one of these novel cell lines (designated clone 2) was selected for all further experiments (Fig 1c). Western blot analyses of culture supernatants from this cell line at passage 11 revealed secretion of about 83.7 ±6.8ng CNTF per 10^5^ cells in 24 hours (mean ±SEM from three independent experiments). Similar amounts of CNTF were found in culture supernatants from passage 19, suggesting stable expression of the cytokine in this cell line. Clonal NS cell lines for control experiments were generated by transducing wild-type NS cells with the lentiviral vector pCAG-IRES-tdTomato/BSD (Fig 1b). Western blot analyses of these control-NS cell clones revealed no detectable amounts of CNTF in the culture supernatants (Fig 1c).

**Fig 2:**
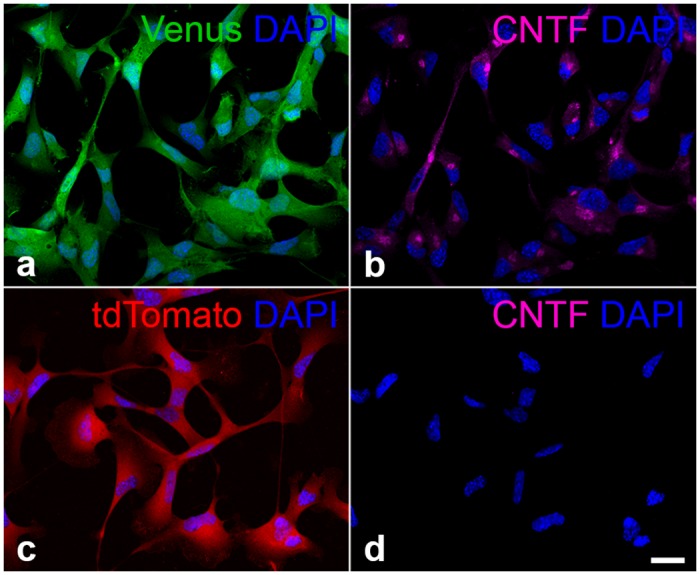
Expression of CNTF and the reporter genes in clonal CNTF-NS and control-NS cell lines. All cells in the clonal CNTF-NS cell line expressed the reporter gene Venus (a) and showed CNTF-immunoreactivity in the perinuclear region (b). Control-NS cells, in comparison, expressed the reporter gene tdTomato (c) but lacked detectable expression of the cytokine (d). CNTF, ciliary neurotrophic factor; DAPI, 4’,6-diamidino-2-phenylindole. Bar in d (for a-d): 20 μm.

Immunocytochemical analyses of undifferentiated NS cells revealed co-expression of CNTF (Fig 2b) and the fluorescent reporter protein Venus (Fig 2a) in all cells of the CNTF-NS cell line. Cells in the control-NS cell line, in comparison, expressed the reporter gene tdTomato (Fig 2c) but no detectable levels of the cytokine (Fig 2d). Moreover, CNTF-immunoreactivity was detectable at similar intensities in the CNTF-NS cell line for at least 23 passages (higher passages were not analyzed), in line with the data obtained by Western blot analyses.

**Fig 3:**
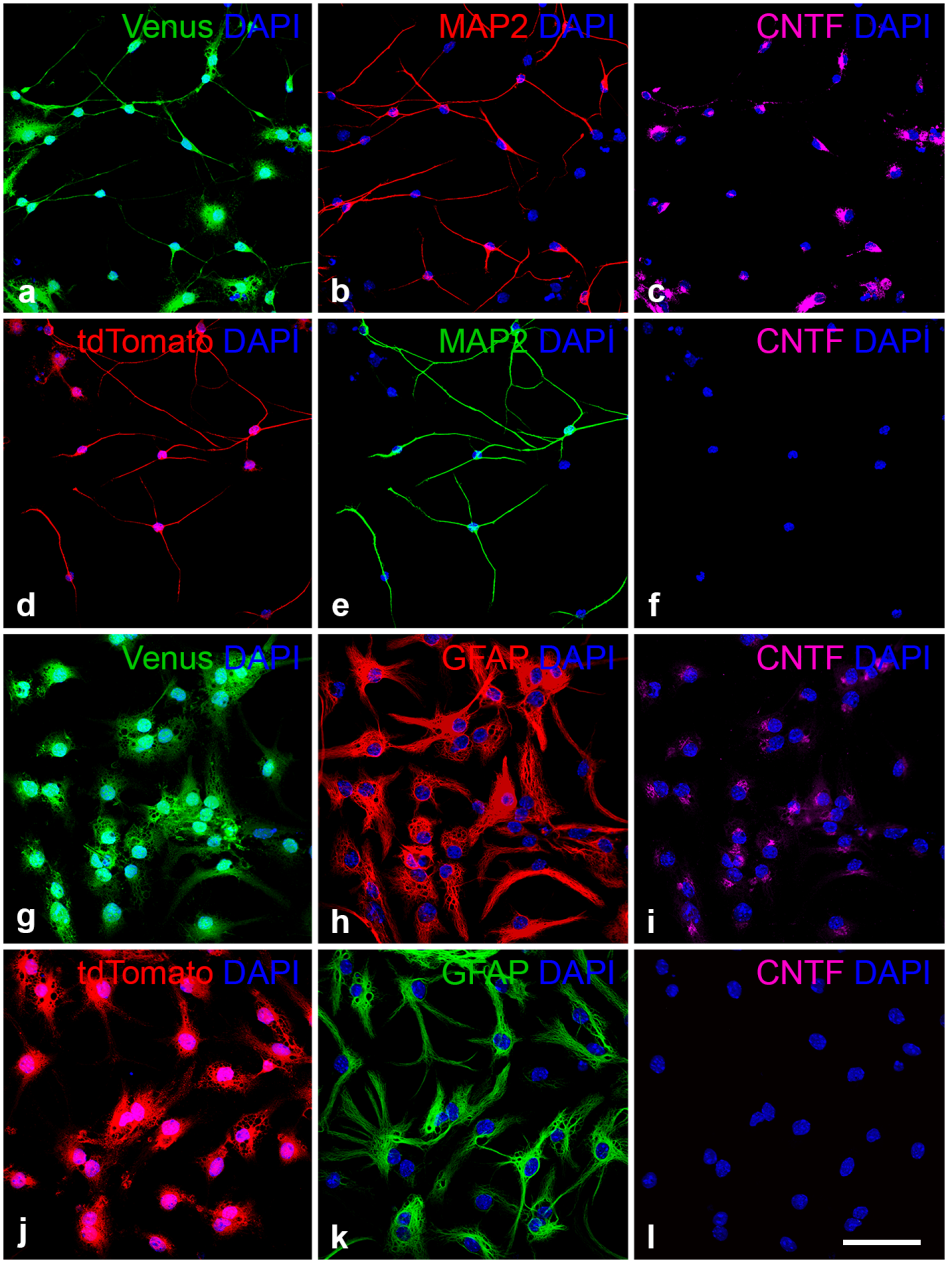
CNTF and reporter gene expression in neural cell types derived from CNTF-NS cells and control-NS cells *in vitro*. CNTF-NS (a-c, g-i) and control-NS cells (d-f, j-l) were differentiated into neurons (a-f) or astrocytes (g-l). Note that all MAP-2-positive neurons (b) and GFAP-positive astrocytes (h) derived from CNTF-NS cells co-expressed the reporter gene Venus (a, g) and CNTF (c, i). Neurons (e) and astrocytes (k) derived from control-NS cells, in comparison, expressed the reporter gene tdTomato (d, j) but no detectable levels of the cytokine (f, l). CNTF, ciliary neurotrophic factor; DAPI, 4’,6-diamidino-2-phenylindole; GFAP, glial fibrillary acidic protein; MAP2, microtubule-associated protein 2. Bar in l (for a-l): 50 μm.

To analyze transgene expression in neural cell types derived from CNTF-NS or control-NS cells *in vitro*, clonal cell lines were differentiated into nerve cells (Fig 3a–3f) or astrocytes (Fig 3g–3l) using directed differentiation protocols. Expression of CNTF in these cultures was analyzed seven days after induction of differentiation. While all MAP-2-positive neurons (Fig 3a–3c) and GFAP-positive astrocytes (Fig 3g–3i) derived from CNTF-NS cells co-expressed the reporter gene Venus and the cytokine, neurons (Fig 3d–3f) and astrocytes (Fig 3j–3l) derived from control-NS cells expressed the reporter gene tdTomato but no detectable levels of CNTF.

### Analysis of intravitreally grafted CNTF-NS and control-NS cells *in vivo*

**Fig 4:**
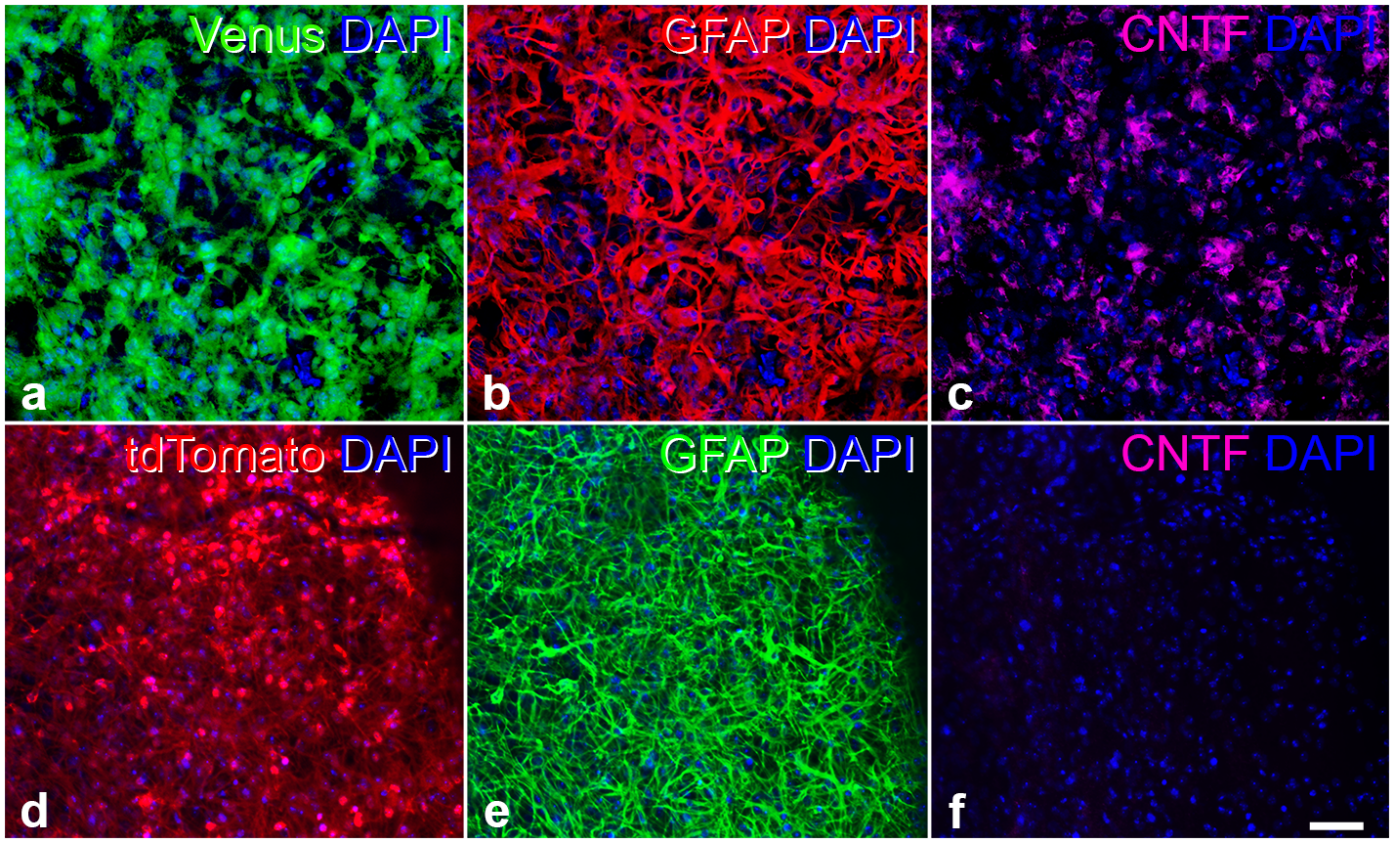
Characterization CNTF-NS and control-NS cells six weeks after intravitreal transplantation into *nclf* mice. Six weeks after intravitreal transplantation, CNTF-NS (a-c) and control-NS cells (d-f) were identified in the host eyes by their expression of the fluorescent reporter proteins Venus (a) and tdTomato (d), respectively. Both, Venus-positive CNTF-NS cells and tdTomato-positive control-NS cells were attached to the posterior poles of the lenses where they were mainly differentiated into GFAP-positive astrocytes (b, e). Expression of CNTF was detectable in astrocytes derived from CNTF-NS cells (c), but not in astrocytes derived from control-NS cells (f). CNTF, ciliary neurotrophic factor; DAPI, 4’,6-diamidino-2-phenylindole; GFAP, glial fibrillary acidic protein. Bar in f (for a-f): 50 μm.

Six weeks after intravitreal transplantation into two weeks old *nclf* mice, grafted CNTF-NS and control-NS cells were identified in the recipient eyes by their expression of the fluorescent reporter proteins Venus (Fig 4a) and tdTomato (Fig 4d), respectively. Both CNTF-NS and control-NS cells had formed dense cell layers that were attached to the posterior poles of the lenses (Fig 4, S1 Fig) or to the vitreal surface of the host retinas (S1 Fig). Formation of tumors or integration of donor cells into the host retinas (S1 Fig) was not observed. Furthermore, we found that 2.1% ±0.36% (mean ± SEM; 6 eyes) of the grafted CNTF-NS cells and 1.8% ±0.38% (6 eyes) of the grafted control-NS cells expressed Ki-67 one week after the transplantation. In comparison, no Ki-67-positive donor cells were detectable six weeks after transplantation of CNTF-NS or control-NS cells (6 eyes for each cell population). The vast majority of the transplanted CNTF-NS and control-NS cells were identified as GFAP-positive astrocytes (Fig 4b and 4e). A few grafted NS cells were differentiated into ß-tubulin III-positive nerve cells, while differentiation of donor cells into myelin-basic protein-positive oligodendrocytes was not observed. Of note, immunocytochemical analyses revealed robust expression of CNTF in CNTF-NS cell-derived astrocytes for at least six weeks after transplantation (Fig 4c), the latest post-transplantation time point analyzed. Donor cells derived from control-NS cells, in comparison, lacked detectable expression of the cytokine (Fig 4f).

### Intravitreally grafted CNTF-NS cells attenuate photoreceptor loss in the *nclf* mouse

**Fig 5:**
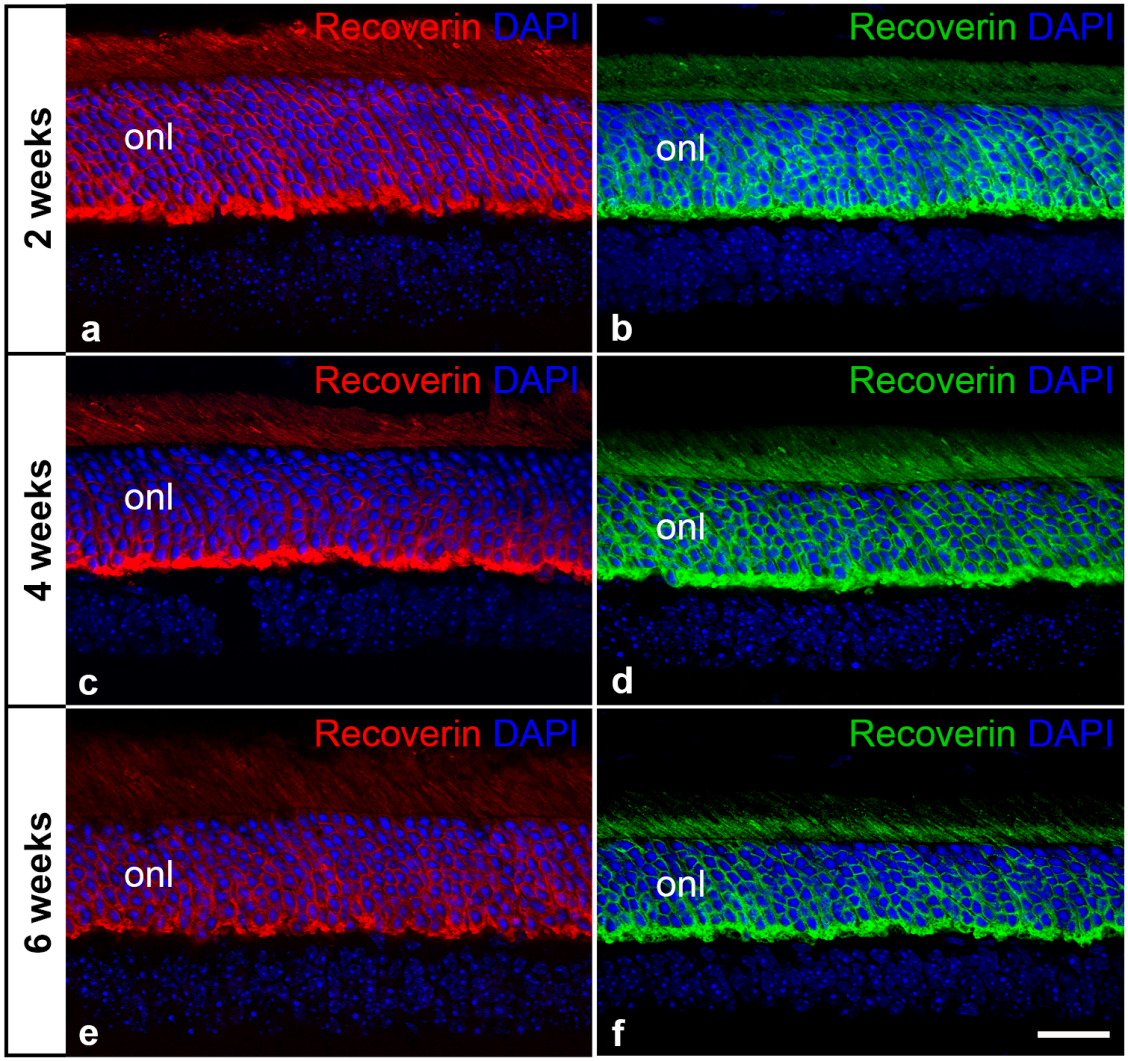
Intravitreally grafted CNTF-NS cells attenuate photoreceptor degeneration in *nclf* mice. A CNTF-NS cell clone was grafted into one (a, c, e) and a control-NS cell clone into the contralateral eye (b, d, f) of 14 days old *nclf* mice. Central retinal sections were stained with anti-recoverin antibodies and DAPI two (a, b), four (c, d) and six (e, f) weeks after transplantation. Note the thicker outer nuclear layer (onl) of CNTF-treated retinas when compared to control retinas at all post-transplantation time points. DAPI, 4’,6-diamidino-2-phenylindole; onl, outer nuclear layer. Bar in f (for a-f): 50 μm.

CNTF-NS cells were intravitreally grafted into 14 days old *nclf* mice, and the neuroprotective effect of the modified cells on photoreceptors was analyzed two, four and six weeks after transplantation (Fig 5a, 5c and 5e). Intravitreal injections of control-NS cells into the contralateral eye of each animal served as a control (Fig 5b, 5d and 5f). Analyses of central (i.e. in the plane of the optic disc) retinal sections that were stained with anti-recoverin antibodies and DAPI consistently revealed a thicker outer nuclear layer of the CNTF-treated retinas when compared to the contralateral control retinas of the same animals at all post-transplantation time points (Fig 5; S2 and S3 Figs). Importantly, this neuroprotective effect on photoreceptor cells was not regionally restricted but evident in all regions of CNTF-treated retinas (S2 and S3 Figs). Adverse effects of the grafted CNTF-NS or control-NS cells on the general histology of the recipient retinas were not observed (Fig 5; S1 and S2 Figs), with the exception of some small-sized retinal folds in a fraction of the CNTF-treated retinas.

**Fig 6:**
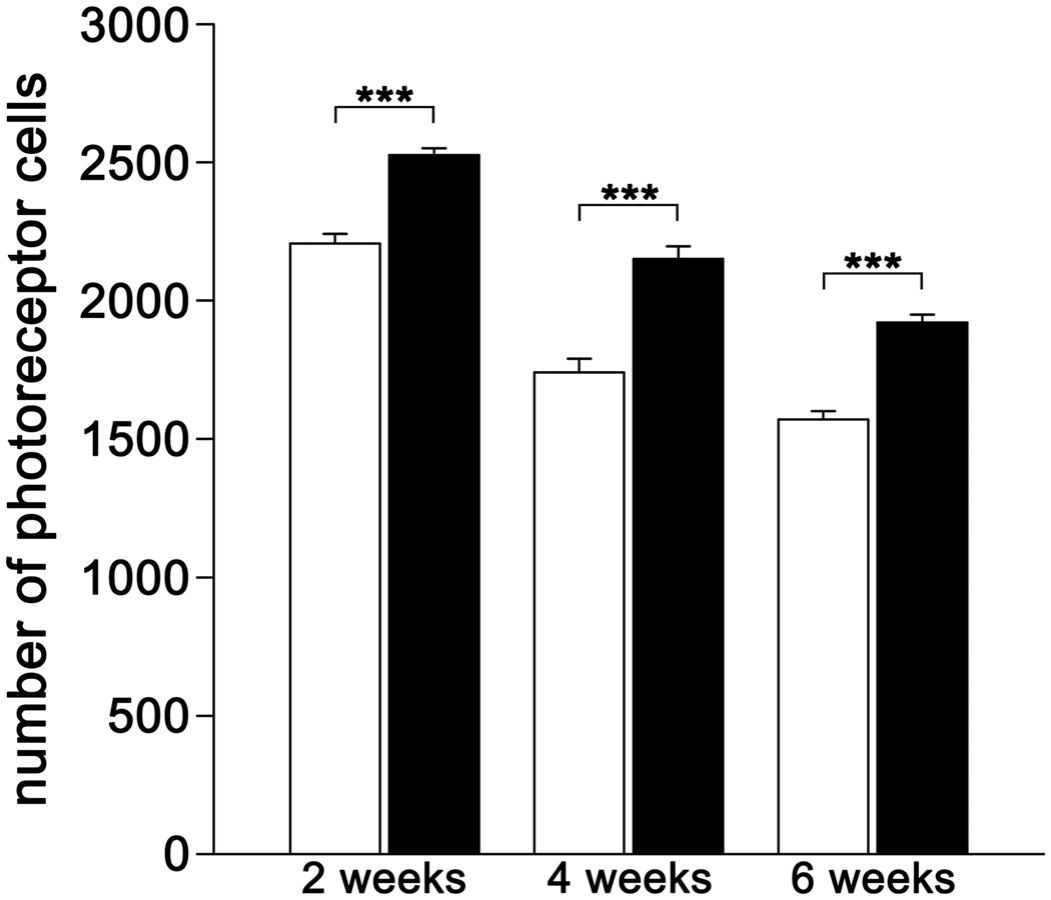
Photoreceptor numbers in eyes of *nclf* mice with grafted CNTF-NS or control-NS cells at different post-transplantation time points. A CNTF-NS and a control-NS cell line were intravitreally grafted into 14 days old *nclf* mice and photoreceptor numbers were determined in central retinal sections at six defined positions two, four and six weeks after transplantation. Note that CNTF-treated eyes contained significantly more photoreceptors (filled bars) than the contralateral eyes with grafted control-NS cells (open bars) at all post-transplantation time points. Each bar represents the mean value (±SEM) from six retinas. ***, p<0.001 (Newman-Keuls post hoc test after the mixed two-way ANOVA).

Determination of photoreceptor numbers in six defined retinal areas located at positions that corresponded to 25%, 50% and 75% of the distance between the optic disc and the periphery of the nasal and temporal retina confirmed a significant protection of photoreceptor cells by the grafted CNTF-NS cells. Two weeks after transplantation, we counted 2,526.2 ±24.8 (mean ± SEM) photoreceptors in the eyes with grafted CNTF-NS cells, compared to 2,206.3 ±34.8 photoreceptors in the contralateral control eyes with grafted control-NS cells (p<0.001 according to the mixed two-way ANOVA test; Fig 6). At four and six weeks after transplantation, CNTF-treated retinas contained 2,150.5 ±45.9 and 1,921.0 ±29.8 photoreceptors, while the contralateral control retinas contained 1,740.2 ±49.5 and 1,570.0 ±29.8 photoreceptors, respectively (p<0.001 for both post-transplantation time points). CNTF-treated retinas thus contained 14.5%, 23.6% and 22.4% more photoreceptor cells than the control retinas two, four and six weeks after the transplantation, respectively (Fig 6). Untreated retinas from one month old wild-type mice (n = 6) were analyzed for comparison and contained 3,174.7 ±60.0 photoreceptor cells.

## Discussion

The neuronal ceroid lipofuscinoses (NCLs) comprise a genetically heterogeneous group of neurodegenerative lysosomal storage disorders that are characterized by intracellular accumulation of autofluorescent material. Dementia, epilepsy and motor deterioration are among the characteristic symptoms of these fatal disorders. Visual impairment due to retinal degeneration is another typical symptom of most NCL forms [1, 3, 4, 6, 7]. For NCL forms caused by mutations in genes encoding soluble lysosomal enzymes, enzyme replacement therapy is among the strategies that are being explored as a potential treatment option. Therapeutic options for NCL forms caused by mutations in genes encoding transmembrane proteins, in comparison, are more limited [20–23, 32].

In the present study, we evaluated whether a sustained cell-based intraocular delivery of a neurotrophic factor ameliorates neurodegeneration in the retina of the *nclf* mouse, an animal model of CLN6 disease that harbors a frameshift mutation in a gene encoding a transmembrane protein [9, 10] of the endoplasmic reticulum (ER) [11]. To this aim, we generated a clonal NS cell line with high expression levels of a secretable variant of mouse CNTF and the fluorescent reporter protein Venus by repeated lentiviral transductions and subsequent clonal expansions. When these cells were grafted into the vitreous cavity of *nclf* mice, they rapidly stopped proliferating and preferentially differentiated into astrocytes that were attached to the posterior surface of the lenses and the vitreal surface of the retinas, similar to our observations in *Pde6b* ^*rd1*^ or *Pde6b* ^*rd10*^ mutant mice [31] and a mouse optic nerve crush model [33]. Integration of control-NS or CNTF-NS cells into the host retinas was not observed over the six weeks post-transplantation time period. The latter finding is in contrast to reports that have shown wide-spread integration of intravitreally grafted neural stem/progenitor cells into developing or dystrophic retinas [24, 34–37]. Intrinsic differences between the neural stem/progenitor cells used in these studies and adherently cultivated NS cells which represent a pure population of symmetrically dividing tripotent neural stem cells [38–40], or the time point of NS cell transplantation prior to the onset of retinal degeneration in the *nclf* mutant [17] may account for these apparently discrepant observations.

Importantly, the NS cell-derived astrocytes survived in the vitreous cavity, stably expressed CNTF and the reporter gene, and significantly delayed the degeneration of photoreceptor cells over a time period of six weeks, the longest post-transplantation interval analyzed. These results are in line with our previous findings in a mouse optic nerve crush model where we observed survival of intravitreally grafted NS cells, stable expression of the cytokine in the donor cells and significant neuroprotective effects on axotomized retinal ganglion cells over a period of four months after cell transplantation [33]. Together, these data suggest that intravitreal transplantations of lentivirally modified NS cells may serve as a useful strategy for preclinical studies aimed at analyzing the long-term effects of cell-based neuroprotective approaches in mouse models of degenerative retinal disorders.

Of note, the protective effect of the CNTF-NS cells on photoreceptor cells in the *nclf* mouse was not regionally restricted but evident in all regions of the experimental retinas, as may be expected for a neuroprotective factor that is continuously released into the vitreous fluid from where it enters the dystrophic retina. Furthermore, we observed no adverse effects of the grafted control-NS cells on the general morphology of the host retinas. However, we found some small-sized retinal folds in restricted regions of a fraction of *nclf* retinas from eyes that had received CNTF-NS cell grafts. The formation of retinal folds has also been observed after repeated intravitreal injections of the CNTF analogue axokine in healthy or dystrophic retinas of cats [41] and in a mouse optic nerve crush model after intravitreal injections of CNTF-secreting neural stem cells [33]. Retinal folds thus appear to be among the complications associated with a sustained intraocular delivery of high doses of CNTF, such as a dysregulation of various genes including some encoding components of the phototransduction cascade and negative effects on visual function in a dose-dependent and reversible manner as measured by electroretinogram recordings [42–46].

Attenuation of photoreceptor degeneration after intraocular transplantations of cells genetically modified to secrete growth factors or cytokines that exert neuroprotective effects on photoreceptor cells, such as glial cell line-derived neurotrophic factor (GDNF), brain-derived neurotrophic factor (BDNF), neurotrophin-4 or CNTF, has also been observed in several other animal models of retinal degeneration, including the Royal College of Surgeon (RCS) rat [47, 48], the S334ter rat [49–51], a sodium iodate-induced mouse model of photoreceptor cell loss [52], the *rcd1* canine model [50] and the *Pde6b* ^*rd1*^ and *Pde6b* ^*rd10*^ mouse [31]. These reports together with the present study demonstrate that intraocular transplantations of genetically engineered cells represent a promising strategy to achieve a sustained administration of neuroprotective factors to the dystrophic retina. In fact, the therapeutic potential of a cell-based delivery of CNTF is currently being explored in human patients with retinitis pigmentosa or geographic atrophy [28] using intravitreal implants of a genetically modified and encapsulated human retinal pigment epithelial (RPE) cell line, indicating the potential relevance of cell-based neuroprotective approaches for clinical applications.

Dietary supplementation with the naturally occurring anti-inflammatory compounds docosahexaenoic acid (DHA) and curcumin has recently been evaluated as another strategy to slow down the progression of retinal degeneration in the *nclf* mouse [18]. DHA and curcumin have both been shown to reduce the production of nitric oxide and the expression of pro-inflammatory cytokines in microglial cells [53–56]. Analyses of retinas from DHA- and curcumin-treated animals revealed that the strong reactive microgliosis normally accompanying retinal degeneration in the *nclf* mutant was strongly diminished, as indicated by the significantly decreased numbers of amoeboid microglial cells. Interestingly, dietary supplementation with DHA and curcumin also delayed deterioration of visual function, while preservation of retinal structure was only observed in DHA-treated but not in curcumin-treated *nclf* mutants [18].

The motor neuron degeneration (*mnd*) mouse represents a naturally occurring animal model of CNL8 disease [57]. Similar to the *nclf* mouse, the *mnd* mouse harbors a mutation in a gene encoding a transmembrane protein of the ER [58, 59] and displays progressive apoptotic degeneration of photoreceptor cells [57, 60–62]. A recent study has analyzed the fate of intravitreally grafted neuralized mouse ES cells in this mutant, and observed wide-spread integration of the donor cells into the *mnd* retinas, where most of the cells acquired a neuronal phenotype. Furthermore and more interestingly, lysosomal storage bodies in *mnd* retinas were significantly reduced in size and fewer in number, and the loss of photoreceptor cells was significantly delayed in retinal regions with integrated donor cells when compared to regions of the same retinas that were devoid of donor cells or to sham-injected control retinas [24]. While the mechanisms by which the grafted cells exerted their neuroprotective effects in this mouse model of CNL8 disease remain to be elucidated, other studies have also observed amelioration of photoreceptor degeneration and partial preservation of visual function after intraocular transplantations of non-modified cell types, including such diverse cell types as neural progenitor cells [47, 63–65], bone marrow-derived stem cells [66–69] and Schwann cells [70]. In most studies, the neuroprotective effects have been attributed to the release of endogenously expressed growth factors and cytokines from the non-modified donor cells [47, 63, 68], or to the induction of neuroprotective factors in the recipient retinas by the grafted cells [65, 69]. Endogenous expression of neuroprotective factors known to rescue photoreceptor cells from degeneration, such as BDNF, FGF-2 or CNTF, in human neural progenitor cells and mesenchymal stem cells is in line with this view [47, 63, 68]. Although not specifically addressed in the present study, non-modified NS cells exerted no neuroprotective effects on photoreceptor cells in the *Pde6b* ^*rd1*^ and *Pde6b* ^*rd10*^ mouse [31], or on retinal ganglion cells in a mouse model of optic nerve injury [33]. To understand these apparently contradictory results, we analyzed non-modified NS cells for the expression of selected growth factors and cytokines by immunocytochemistry and Western blot analyses of culture supernatants, and found no detectable expression levels of BDNF, GDNF or CNTF. Furthermore, the ability of a certain cell population to attenuate retinal degeneration may also depend on the specific pathomechanisms ultimately leading to the death of photoreceptor cells and may therefore differ in different animal models of retinal degeneration, as indicated by recent work on the RCS rat. Photoreceptor cells in this mutant die because phagocytosis of photoreceptor outer segments by RPE cells is impaired due to a mutation in the *Mertk* gene. Based on the observation that subretinally grafted human neural progenitor cells are capable to phagocytose shed outer segments in this rat mutant, it has been suggested that the non-modified neural progenitor cells confer their neuroprotective activity on photoreceptors in this animal model, at least in part, by functionally replacing the dysfunctional RPE [63, 71].

The therapeutic potential of a sustained cell-based administration of CNTF is currently being evaluated in patients with retinitis pigmentosa or geographic atrophy using intravitreal implants of an encapsulated human RPE cell line genetically modified to secrete this cytokine [72–76]. Analyses of the efficacy of this so-called encapsulated cell technology in mouse models of retinal degeneration are impeded by the large size of the encapsulated cell implants. The cell-based neuroprotective approach described in the present study mimics some aspects of the encapsulated cell technology, in that modified and intravitreally located cells provide a continuous supply of a neuroprotective factor to a dystrophic retina. We therefore suggest that this NS cell-based approach represents a useful methodology for preclinical studies aimed at analyzing the therapeutic potential of a cell-based intravitreal delivery of neurotrophic factors on retinal structure and function in mouse models of photoreceptor loss.

## Supporting Information

S1 Fig Localization of intravitreally grafted CNTF-NS cells in eyes of *nclf* mice. Analysis of eyes from *nclf* mice six weeks after intravitreal transplantation of CNTF-NS cells revealed the presence of Venus-positive donor cells (arrowheads in a) that were attached to the vitreal surface of the retinas. Integration of Venus-positive cells into the host retinas was not observed. Donor cells were also found on the posterior surfaces of the lenses (arrowheads in b). DAPI, 4’,6-diamidino-2-phenylindole; gcl, ganglion cell layer; inl, inner nuclear layer; onl, outer nuclear layer. Bar in f (for a-f): 50 μm.
(TIF)

S2 Fig Morphology of host retinas after intravitreal transplantations of NS cells. CNTF-NS cells (a) and control-NS cells (b) were grafted into the vitreous cavity of 14 days old *nclf* mice, and retinas were analyzed six weeks after transplantation. Note the increased thickness of the outer nuclear layer (onl) in all regions of the CNTF-treated retina (a) when compared to the contralateral control retina (b). Adverse effects of the grafted cells on the general morphology of the host retinas were not detectable (a, b). Shown are overviews of the entire nasal half of a CNTF-treated and a contralateral control retina in central retinal sections. DAPI, 4’,6-diamidino-2-phenylindole; ipl, inner plexiform layer; ON, optic nerve. Bar in b (for a and b): 200 μm.
(TIF)

S3 Fig Thickness of the outer nuclear layer in CNTF-treated and control retinas. CNTF-NS cells were grafted into one and control NS-cells into the contralateral eye of 14 days old *nclf* mice, and the thickness of the outer nuclear layer was determined at 18 equally spaced positions between the peripheral margins of the nasal and temporal retina two (a), four (b) and six (c) weeks after transplantation. The outer nuclear layer was consistently thicker in CNTF-treated (red circles) when compared to control treated eyes (blue squares) at all post transplantation time points. Each symbol represents the mean value (±SEM) from six retinas, *: p<0.05; **:p<0.01; ***p<0.001 according to the Student’s t-test for paired samples. onh, optic nerve head.
(TIF)
