# Supplementary figures and images for: Sustained Neural Stem Cell-Based Intraocular Delivery of CNTF Attenuates Photoreceptor Loss in the nclf Mouse Model of Neuronal Ceroid Lipofuscinosis
Source: PLoS One. 2015 May 20;10(5):e0127204. doi: 10.1371/journal.pone.0127204 (PMC4439090; doi:10.1371/journal.pone.0127204)

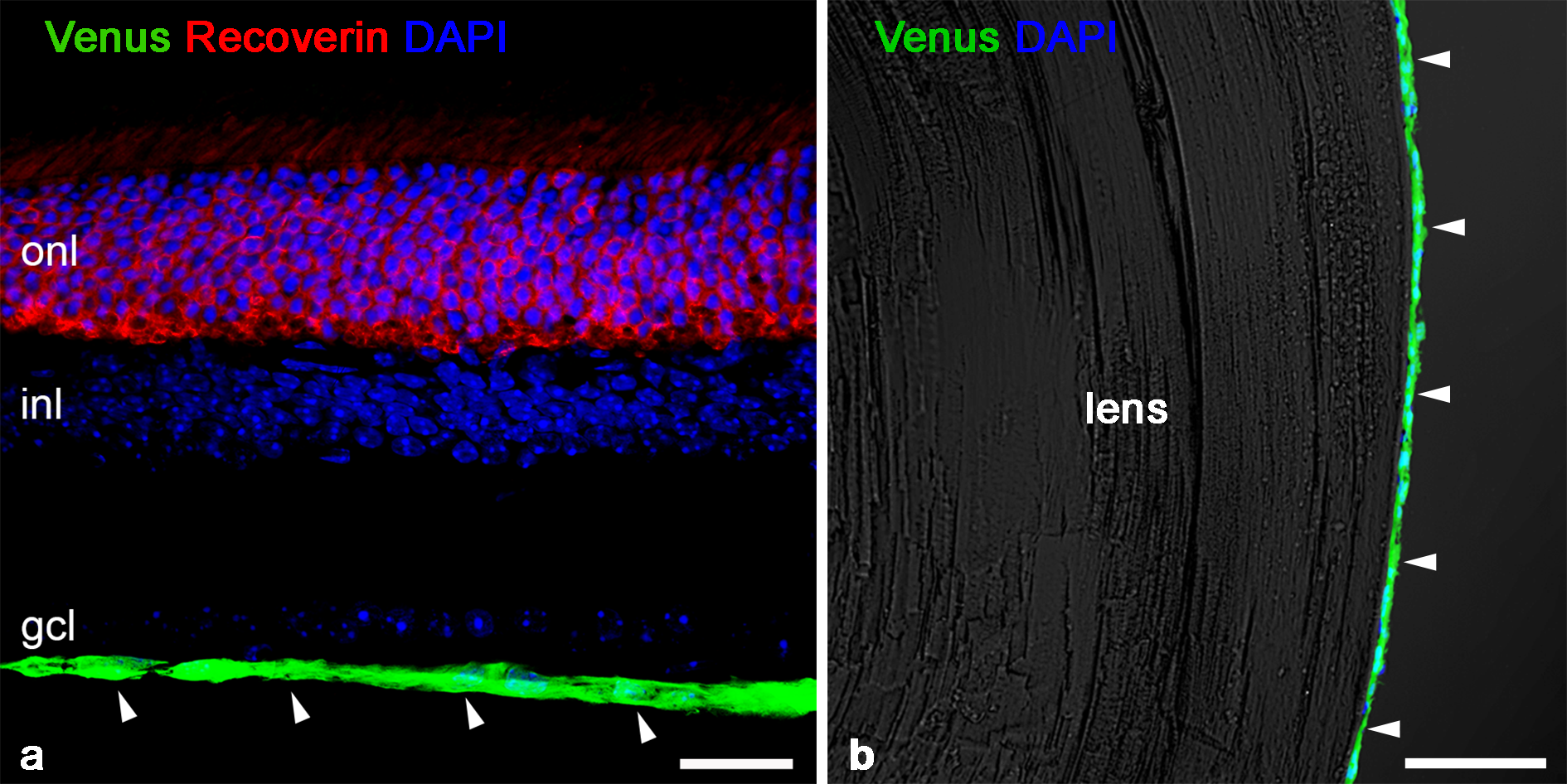

Supplement: S1 Fig — Analysis of eyes from nclf mice six weeks after intravitreal transplantation of CNTF-NS cells revealed the presence of Venus-positive donor cells (arrowheads in a) that were attached to the vitreal surface of the retinas. Integration of Venus-positive cells into the host retinas was not observed. Donor cells were also found on the posterior surfaces of the lenses (arrowheads in b). DAPI, 4’,6-diamidino-2-phenylindole; gcl, ganglion cell layer; inl, inner nuclear layer; onl, outer nuclear layer. Bar in f (for a-f): 50 μm. (TIF) [file pone.0127204.s001.tif]

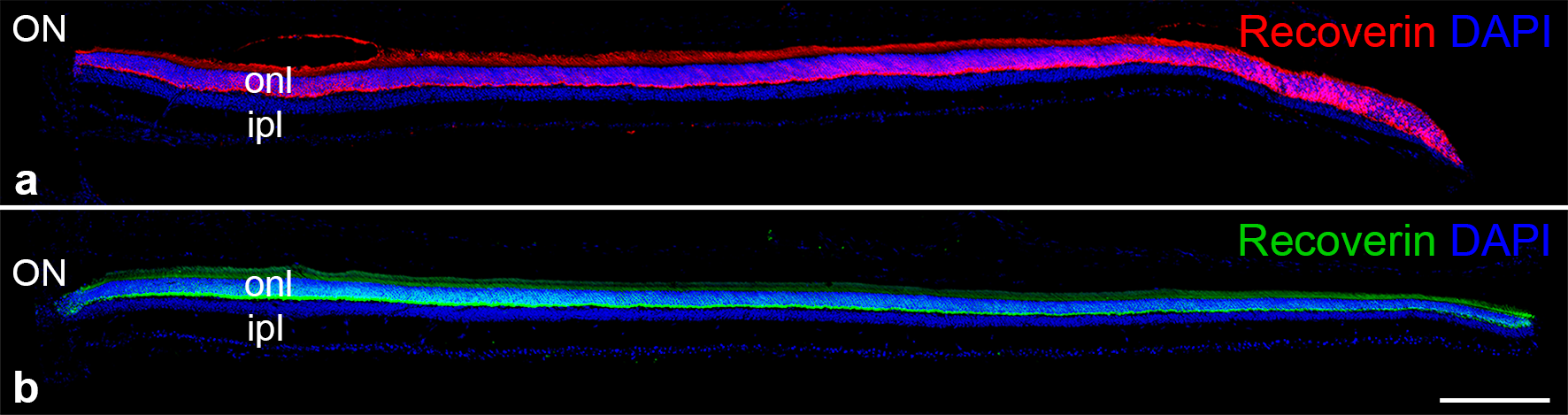

Supplement: S2 Fig — CNTF-NS cells (a) and control-NS cells (b) were grafted into the vitreous cavity of 14 days old nclf mice, and retinas were analyzed six weeks after transplantation. Note the increased thickness of the outer nuclear layer (onl) in all regions of the CNTF-treated retina (a) when compared to the contralateral control retina (b). Adverse effects of the grafted cells on the general morphology of the host retinas were not detectable (a, b). Shown are overviews of the entire nasal half of a CNTF-treated and a contralateral control retina in central retinal sections. DAPI, 4’,6-diamidino-2-phenylindole; ipl, inner plexiform layer; ON, optic nerve. Bar in b (for a and b): 200 μm. (TIF) [file pone.0127204.s002.tif]

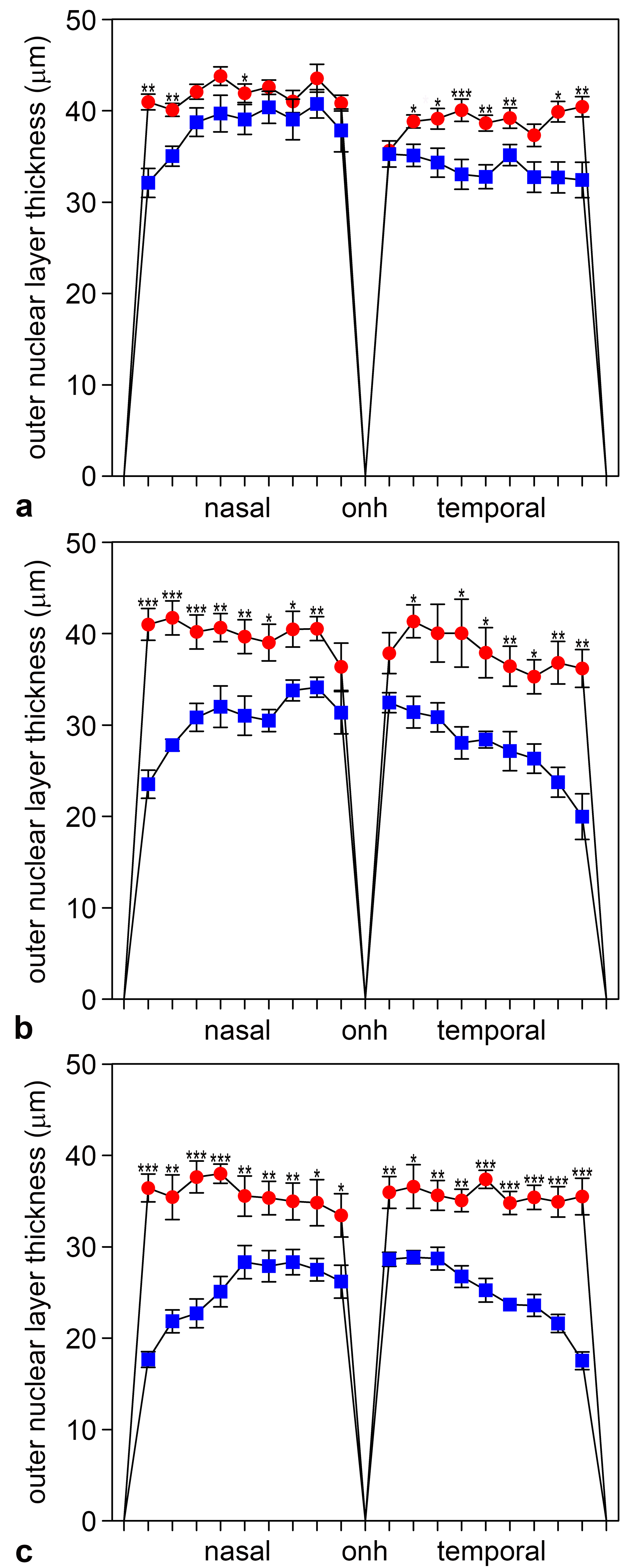

Supplement: S3 Fig — CNTF-NS cells were grafted into one and control NS-cells into the contralateral eye of 14 days old nclf mice, and the thickness of the outer nuclear layer was determined at 18 equally spaced positions between the peripheral margins of the nasal and temporal retina two (a), four (b) and six (c) weeks after transplantation. The outer nuclear layer was consistently thicker in CNTF-treated (red circles) when compared to control treated eyes (blue squares) at all post transplantation time points. Each symbol represents the mean value (±SEM) from six retinas, *: p<0.05; **:p<0.01; ***p<0.001 according to the Student’s t-test for paired samples. onh, optic nerve head. (TIF) [file pone.0127204.s003.tif]
